# Influences of Temperature on Development and Survival, Reproduction and Growth of a Calanoid Copepod (*Pseudodiaptomus dubia*)

**DOI:** 10.1100/tsw.2009.96

**Published:** 2009-09-01

**Authors:** Changling Li, Xiaoxia Luo, Xianghu Huang, Binhe Gu

**Affiliations:** Department of Aquaculture, Fishery College, Guangdong Ocean University, Xiashan, Zhanjiang, Guangdong, China

**Keywords:** copepods, growth rate, larval development, Pseudodiaptomus dubia, temperature

## Abstract

*Pseudodiaptomus dubia* is a calanoid copepod that is distributed widely in the estuarine-coastal waters of Asia and is a dominant copepod in the shrimp grow-out ponds in southern China. A laboratory culture experiment was conducted to evaluate the influences of water temperature on larval development, survival, and reproduction. Results indicate that within a temperature range from 15 to 35°C, larval development increases as the temperature increases. The water temperature for optimal larval survival rate ranges from 20 to 35°C. Longevity and egg hatching time decrease as the temperature increases from 20 to 35°C. Total fecundity and reproduction frequency increase as the water temperature increases, with the maximum at 30°C. Fecundity and reproduction frequency decrease when the temperature exceeds 30°C. Intrinsic growth rate (r_m_) ranges from 0.168 to 0.195 at 25 to 30°C; net reproduction rate (R_0_) and finite growth rate (?) are 163 to 264 and 1.183 to 1.215, respectively, when the temperature is greater than 20 and 35°C; population doubling time (t) varies from 3.556 to 4.128 days at temperatures less than 20 and 35°C. Population generation time (T) is negatively correlated with temperature, with the optimal population growth rate at 25 to 30°C.

### INTRODUCTION

Temperature is a vital factor that influences mortality[1], instar duration[2,3], molting rate[4,5], and reproduction of copepods[5,6]. Within a suitable range, increases in temperature will increase egg production and growth rate, and shorten reproduction cycle and hatching time. Different species that live in different geographical regions may have specific requirements for temperature. Temperature affects the energy storage and hormone production that control larval development. Molting will not take place until levels of energy and hormone exceed a threshold[7].

*Pseudodiaptomus dubia*[8], previously reported as *Schmackeria dubia*[9], is a calanoid copepod that is distributed widely in Asian estuarine-coastal waters and is a dominant copepod in the shrimp grow-out ponds in southern China. Aside from the morphological characteristics[10], little is known about the effects of environmental factors on the ecology of *P. dubia*. Recently, we reported on the responses of feeding and filtering rates of *P. dubia* to the changes in several environmental factors, including water temperature[9]. Here we report the effects of temperature on reproduction, development, and growth. Findings from this study will provide important insights into the thermal condition that favors mass production of *P. dubia*.

### MATERIALS AND METHODS

This experiment was conducted at the Guangdong Ocean University, Guangdong, China between April and June 2006. Previously, we reported the effects of several environmental variables on the filtering and feeding rates of adult *P. dubia*[9]. The objective of the current study was to assess the effects of various water temperatures on the growth and reproduction of *P. dubia* at different development stages.

#### Experimental Animals

*P. dubia* were collected from a shrimp farm (water temperature, 30ºC; salinity, 27 ppt) in Zhanjiang, southern China. In the laboratory, the copepods were raised at a water temperature between 28 and 30ºC, a salinity of 27 ppt, and a light intensity of 160 to 240 µmol $m^{-2}$ $s^{-1}$[9]. After the copepods were acclimated to the culture conditions, healthy and energized egg-carrying females were carefully transferred with a pipe to the culture media. Their offspring were used as the experiment animals.

A unicellular golden alga (*Isochrysis zhanjiangensis*) and a diatom (*Chaetoceros müelleri*) provided by the Algal Culture Research Laboratory of Guangdong Ocean University (Zhanjiang, China) were used as food sources. Natural seawater was filtered using a plankton net (mesh size, 200 µm) and boiled to kill all living organisms. Wide-mouth transparent beakers (125 ml) were used as the culture containers.

#### Larval Selection

Ten milliliters of fresh seawater was added to each of 200 pipettes. Copepods were transferred from the culture media and examined under a microscope. A total of 200 female copepods that were carrying matured egg sacs were then transferred into the 200 pipettes and raised at a water temperature of 30ºC, a light intensity of 200 µmol $m^{-2}$ $s^{-1}$, and a light:dark cycle of 12 h:12 h.

Newly hatched larvae were collected every 2 h; larvae from the time period with the highest hatching rate were transferred to a beaker and used for the experiment.

#### Experimental Design

There were five temperature levels (15, 20, 25, 30, 35ºC), each of which contained 10 replicate groups. Each group within a temperature level consisted of 10 incubation bottles that were further divided into five subgroups, namely A, B, C, D, and E. Each subgroup consisted of two bottles. Each bottle had 125 ml seawater and 10 nauplii added to it. Group A was used to record the development and growth of nauplii and copepodites; Group B was used to record survival rate of nauplii; Group C was used to record survival rate of copepodites. Experimental animals in Groups D and E (four incubation bottles) were used for the reproduction experiment. All animals were fed with *C. müelleri* at an identical concentration of 20 $\times 10^4$ individuals/milliliter. Incubation was conducted at a salinity of 27 ppt, a pH of 8.0, a light intensity of 200 µmol $m^{-2}$ $s^{-1}$, and a light:dark cycle of 12 h:12 h.

### Growth and Survival Rates

The life cycle of *P. dubia* consisted of 12 stages, including six naupliar stages (NI, NII, NIII, NIV, NV, and NVI), five copepodite stages (CI, CII, CIII, CIV, and CV), and the adult stage. Because *P. dubia* is an egg-carrying invertebrate, eggs in the egg sacs develop into NI before the sacs are detached from the females and the NI nauplii are then hatched. NI nauplii develop into NII within 2–4 min of hatching. Therefore, our observation on larval development began at stage NII.

Developmental stages were monitored every 6 h by randomly taking three to four larvae from Group A bottles. The larvae were staged and returned to the same bottles. When nauplii in Group B reached NVI, they were preserved in formalin. The numbers of survival larvae and prosome length were recorded. When the larvae in Group C reached the adult stage, the numbers of survival and prosome length of females and males were recorded. These data were used to calculate growth and survival rates for the naupliar and copepodite stages.

Survival rate (*SR*) was calculated using the following equation:

$$SR = \frac{a_1}{10} \times 100\% \quad (1)$$

where $a_1$ is the live larvae at the end of the incubation. Average growth rate was calculated as

$$\log C = 2.00 \log CL - 5.67 \text{ (nauplii)} \quad (2)$$

$$\log C = 2.87 \log L - 8.03 \text{ (copepodites)} \quad (3)$$

$$G = (C_{i+1} - C_i) / T_i \quad (4)$$

where *CL* and *L* are the prosome length (µm) of nauplii and copepodites, $C_i$ is the weight (µg carbon) in stage *i* larvae, and $T_i$ is the time (days) of stage *i* larvae.

### Reproductivity Experiment

When the adult copepods in Groups D and E started carrying eggs, females and males at a ratio of 1:2 were incubated in six to 10 test tubes containing algal solutions. The hatching rate was recorded every 1–2 days. Incubation was not terminated until all female adults died. These data were used to calculate a reproductivity table that was then used to calculate intrinsic rate of population growth ($r_m$), finite rate of growth (λ), net reproduction rate ($R_0$), and generation time (T).

Life parameters of *P. dubia* were calculated following Shen[11] and Tang et al.[12]:

*Reproduction rate:*

$$R_0 = \Sigma l_x m_x \quad (5)$$

*Generation time:*

$$T = \Sigma x l_x m_x / R_0 \quad (6)$$

*Intrinsic rate of population growth:*

$$r_m = \ln R_0 / T \quad (7)$$

*Finite rate of growth:*

$$\lambda = e^{r_m} \tag{8}$$

*Population doubling time:*

$$t = (\ln 2) / r_m \tag{9}$$

where $x$ is age or time interval, $l_x$ is survival rate at time x, and $m_x$ is birth rate at age x.

All statistical procedures were performed using SPSS software (version 11). Significant tests were performed using the ANOVA procedure. Results were considered significant at $p = 0.05$.

### RESULTS

#### Morphological Development for Nauplii and Copepodites

The major changes in morphology for each stage of nauplii and copepodite are listed in Tables 1 and 2.

**TABLE 1**
**Main Characteristics of Each Stage of Nauplii and Copepodites of *P. dubia***

| Nauplii | No. of Seta on Last Segment of Antenna Prima | Body Length (mm) | Copepodite | No. Prosome Segments | No. Abdominal Segments | Length of Thoracic Segment (mm) |
|---|---|---|---|---|---|---|
| NI | Last segment: 4 | 0.121–0.139 | CI | 3 | 2 | 0.325–0.425 |
| NII | Last segment: 4 | 0.161–0.17 | CII | 4 | 2 | 0.4674–0.5043 |
| NIII | Abdominal: 1<br>Last segment: 4 | 0.190–0.22 | CIII | 5 | 2 | 0.5576–0.63345 |
| NIV | Dorsal: 4<br>Abdominal: 3<br>Last segment: 4 | 0.225–0.25 | CIV | 5 | 3 | 0.79335–0.825 (F)<br>0.6765–0.76875 (M) |
| NV | Dorsal: 6<br>Abdominal: 4<br>Last segment: 4 | 0.271–0.30 | CV | 5 | 3 (F)<br>4 (M) | 0.933043–1.0332 (F)<br>0.7995–0.861 (M) |
| NVI | Dorsal: 8<br>Abdominal: 4<br>Last segment: 4 | 0.305–0.32 | Adult | 4 | 3 (F)<br>4 (M) | 1.0947–1.1931 (F)<br>0.9471–1.04165 (M) |

*Note:* F, female; M, male.

#### Effect of Temperature on Development Time of Larval *P. dubia*

When water temperature ranges from 15 to 35ºC, the development time required for each stage decreases as the temperature increases (Table 2, Figs. 1 and 2). At the nauplius stages, development time is minimal (66 h) at a water temperature of 30 to 35ºC. However, it takes 294 h to complete all of the nauplius stages at 15ºC. At the copepodite stages, it takes 114 h for copepodite stage I to develop into the adult stage at 35ºC, which is 520 h shorter than at 15ºC. The entire larval development time is 180 h at 35ºC and 928 h at 15ºC.

**TABLE 2**
**Development Time (h) of Larvae of *P. dubia* under Different Temperatures**

| Larval Stage | 15°C | 20°C | 25°C | 30°C | 35°C |
|---|---|---|---|---|---|
| NII | 54 | 24 | 18 | 12 | 12 |
| NIII | 60 | 24 | 18 | 12 | 12 |
| NIV | 60 | 32 | 18 | 12 | 12 |
| NV | 60 | 32 | 18 | 12 | 12 |
| NVI | 60 | 32 | 24 | 18 | 18 |
| NII–NVI | 294 | 144 | 96 | 66 | 66 |
| CI | 80 | 30 | 24 | 18 | 18 |
| CII | 112 | 42 | 36 | 24 | 24 |
| CIII | 128 | 48 | 36 | 30 | 24 |
| CIV | 144 | 54 | 42 | 36 | 24 |
| CV | 170 | 66 | 42 | 36 | 24 |
| CI–CV | 634 | 240 | 180 | 144 | 114 |
| Sum (h) | 928 | 384 | 276 | 210 | 180 |

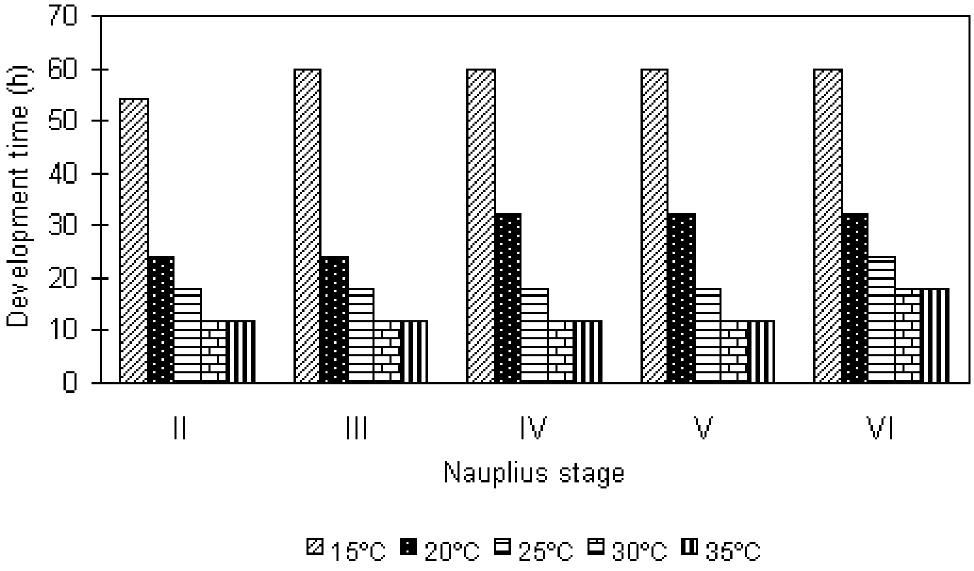

**FIGURE 1**. The development time of nauplii of *P. dubia* under different temperatures.

### Effect of Temperature on the Growth of *P. dubia*

The effect of temperature on prosome length and weight for larvae of the same stage was minor (Tables 3 and 4). This is because prosome length and weight gains were insignificant at the naupliar stage. However, changes in weight at the copepodite stage were significant.

At a temperature range between 15 and 30ºC, the growth rate for larvae was consistently higher than that of larvae grown at a low temperature (Fig. 3 and 4). For example, the growth rate for CV at 35, 30, 25, and 20ºC was 6.35-, 3.72-, 3.05-, and 2.81-fold higher than the growth rate at 15ºC, indicating that at high temperatures, copepodite metabolism accelerated, leading to a higher growth rate.

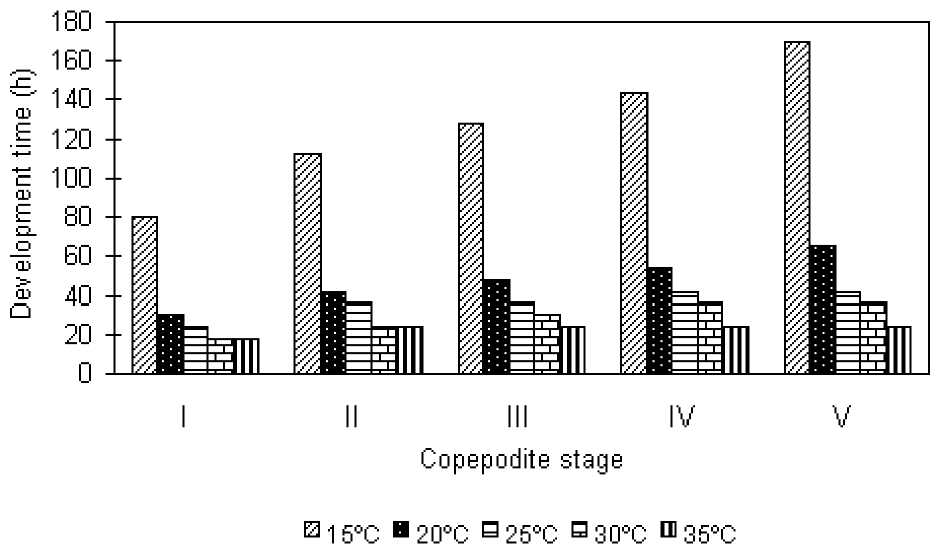

**FIGURE 2.** Development time of copepodites of *P. dubia* under different temperatures.

**TABLE 3**
**Effects of Temperature on Development and Growth of the Naupliar Stages *of P. dubia***

| Instar | Parameter | 15°C | 20°C | 25°C | 30°C | 35°C |
|---|---|---|---|---|---|---|
| NII | Prosome length (μm) | 165 | 170 | 165 | 170 | 170 |
| | Weight (μg C) | 0.0582 | 0.0618 | 0.0582 | 0.0618 | 0.0600 |
| | Development time (days) | 2.25 | 1 | 0.75 | 0.5 | 0.5 |
| | Growth rate (μg C/day) | 0.0201 | 0.0370 | 0.0517 | 0.0723 | 0.0759 |
| NIII | Prosome length (μm) | 220 | 215 | 213 | 214 | 216 |
| | Weight (μg C) | 0.1035 | 0.09883 | 0.097 | 0.0979 | 0.1 |
| | Development time (days) | 2.5 | 1 | 0.75 | 0.5 | 0.5 |
| | Growth rate (μg C/day) | 0.01206 | 0.0295 | 0.0390 | 0.0464 | 0.0346 |
| NIV | Prosome length (μm) | 250 | 245 | 243 | 238 | 234 |
| | Weight (μg C) | 0.1336 | 0.1283 | 0.1262 | 0.1211 | 0.12 |
| | Development time (days) | 2.5 | 1.3333 | 0.75 | 0.5 | 0.5 |
| | Growth rate (μg C/day) | 0.0160 | 0.0206 | 0.0536 | 0.1028 | 0.1035 |
| NV | Prosome length (μm) | 285 | 270 | 279 | 284 | 281 |
| | Weight (μg C) | 0.1737 | 0.1559 | 0.1664 | 0.1724 | 0.17 |
| | Development time (days) | 2.5 | 1.3333 | 0.75 | 0.5 | 0.5 |
| | Growth rate (μg C/day) | 0.0209 | 0.0483 | 0.0774 | 0.0957 | 0.0975 |
| NVI | Prosome length (μm) | 325 | 321 | 324 | 321 | 319 |
| | Weight (μg C) | 0.2258 | 0.2203 | 0.2244 | 0.2203 | 0.22 |
| | Development time (days) | 2.5 | 1.3333 | 1 | 0.75 | 0.75 |
| | Growth rate (μg C/day) | 0.0605 | 0.0603 | 0.0527 | 0.1179 | 0.1108 |

**TABLE 4**
**Effects of Temperature on Development and Growth of Copepodite Stage *of P. dubia***

| Instar | Parameter | 15°C | 20°C | 25°C | 30°C | 35°C |
|---|---|---|---|---|---|---|
| CI | Prosome length (µm) | 420 | 375 | 360 | 380 | 375 |
| | Weight (µg C) | 0.2195 | 0.1596 | 0.1423 | 0.1657 | 0.16 |
| | Development time (days) | 3.3333 | 1.25 | 1 | 0.75 | 0.75 |
| | Growth rate (µg C/day) | 0.04424 | 0.1274 | 0.2000 | 0.2355 | 0.1824 |
| CII | Prosome length (µm) | 504.3 | 479.7 | 492 | 492 | 467 |
| | Weight (µg C) | 0.3669 | 0.3188 | 0.3423 | 0.3423 | 0.3 |
| | Development time (days) | 4.6667 | 1.75 | 1.5 | 1 | 1 |
| | Growth rate (µg C/day) | 0.0587 | 0.1997 | 0.1960 | 0.1647 | 0.1902 |
| CIII | Prosome length (µm) | 615 | 614.225 | 613.45 | 565.8 | 558 |
| | Weight (µg C) | 0.6408 | 0.6682 | 0.6963 | 0.5070 | 0.49 |
| | Development time (days) | 5.3333 | 2 | 1.5 | 1.25 | 1 |
| | Growth rate (µg C/day) | 0.0828 | 0.2133 | 0.3185 | 0.3353 | 0.3510 |
| CIV | Prosome length (µm) | 741.075 | 744.15 | 768.75 | 701.1 | 677 |
| | Weight (µg C) | 1.0822 | 1.0949 | 1.114 | 0.9261 | 0.84 |
| | Development time (days) | 6 | 2.25 | 1.75 | 1.5 | 1 |
| | Growth rate (µg C/day) | 0.0837 | 0.2465 | 0.2687 | 0.4387 | 0.6829 |
| CV | Prosome length (µm) | 848.7 | 861 | 848.7 | 848.7 | 836 |
| | Weight (µg C) | 1.5842 | 1.6495 | 1.5842 | 1.5842 | 1.52 |
| | Development time (days) | 7.0833 | 2.75 | 1.75 | 1.5 | 1 |
| | Growth rate (µg C/day) | 0.1114 | 0.4246 | 0.4517 | 0.5261 | 0.8189 |

*Note:* Body lengths for CIV–CV were based on the body length of males.

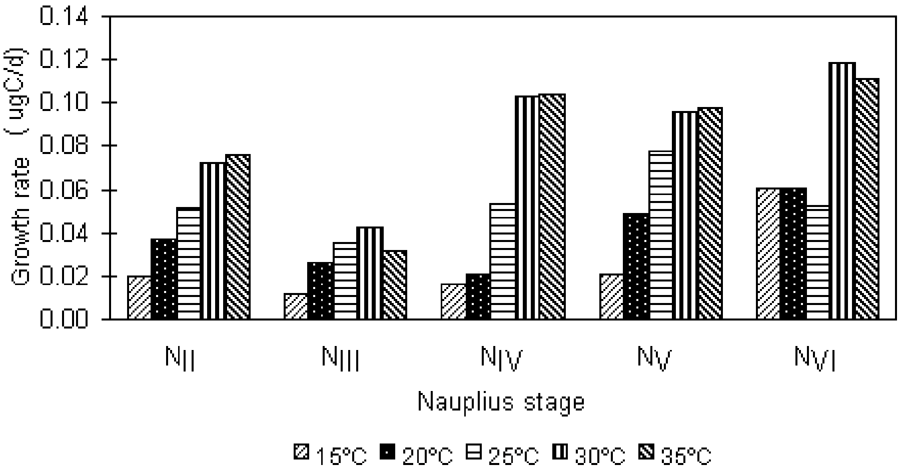

**FIGURE 3.** Effect of temperature on growth rate of *P. dubia* nauplii.

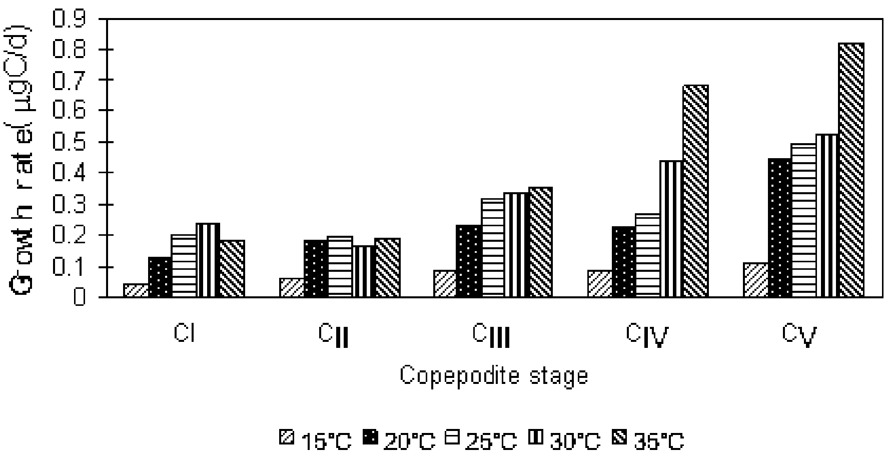

**FIGURE 4.** Effect of temperature on growth rate of *P. dubia* copepodites.

### Effects of Temperature on Larval Survival Rate

The effects of temperature on the survival rate of juvenile *P. dubia* were significant (Fig. 5 and Table 5). Within the suitable range, the larval survival rate at high temperatures was always higher than that at low temperatures. The survival rates for both nauplii and copepodites were highest at a temperature of 30ºC and were 80 and 67.5%, respectively, and decreased when the temperature exceeded 30ºC. The survival rate for nauplii at 30 and 35ºC was significantly higher than at 15 and 20ºC ($p < 0.05$); the survival rate for copepodites at 20 and 35ºC was significantly higher than at 15ºC ($p < 0.05$), with the survival rate at 30ºC 1.45-fold of that at 15ºC. The most suitable growth temperature for *P. dubia* ranged from 20 to 35ºC.

### Effects of Temperature on Reproduction

The life span and hatching time for *P. dubia* decreased as the temperature increased, and were lowest at 35ºC (41 and 1.02 days, respectively), which were about 64 and 60% less than those at 20ºC (Table 6). When the temperature ranged from 20 to 30ºC, the total larval production and reproduction time increased as the temperature increased, but decreased when the temperature exceeded 30ºC. The highest average larval production and reproduction frequency appeared at 30ºC, accounting for 480 individuals/female and 30 times of reproduction, followed at 25ºC. Therefore, the optimal reproduction temperature ranged from 25 to 30ºC.

### Effects of Temperature on Reproduction and Growth Parameters

The intrinsic growth rate, finite growth rate, and net growth rate were 0.195, 1.215, 264, respectively, at 30ºC (Tables 7 and 8), followed by 25°C (0.168, 1.183, and 163) and 35ºC (0.079, 1.082, and 3.3). At temperatures between 25 and 30°C, the population doubling time was 3.556 to 4.128 days, which were less than those at 20 and 35°C. However, the generation time decreased as the temperature increased, with the shortest at 25°C (15.36 days) and longest at 20°C (58.5 days). The optimal temperatures for population growth ranged from 25 to 30°C.

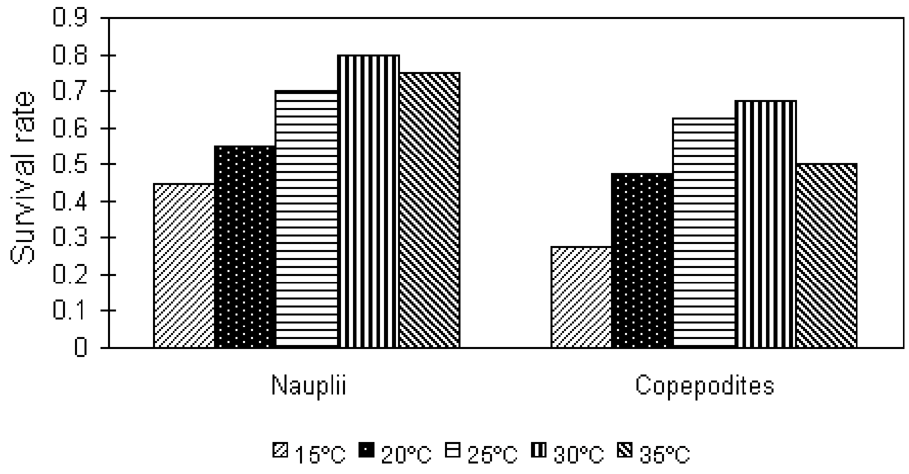

**FIGURE 5.** Effect of temperature on survival rate of *P. dubia.*

**TABLE 5**
**Variance Analysis of Survival Rate of *P. dubia* under Different Temperatures**

| Variance | | SS | *df* | Ms | F | $F_{0.05}$ |
|---|---|---|---|---|---|---|
| Nauplii | Treatment | 0.17 | 4 | 0.0425 | 6.0714* | 3.06 |
| | Error | 0.035 | 5 | 0.007 | | |
| | Total variance | 0.205 | 9 | | | |
| Copepodites | Treatment | 0.388 | 4 | 0.097 | 4.4091* | 3.06 |
| | Error | 0.33 | 5 | 0.022 | | |
| | Total variance | 0.718 | 9 | | | |

* Significant at $p < 0.05$.

**TABLE 6**
**Growth and Production Parameters of *P. dubia* under Different Temperatures**

| Growth/Reproduction Indices | Temperature (℃) | | | |
|---|---|---|---|---|
| | 20 | 25 | 30 | 35 |
| Development stage before hatching (days) | 16 | 11.5 | 8.75 | 7.5 |
| Reproduction stage (day) | 100.2 | 54 | 46.5 | 33.8 |
| Range of longevity (days) | 84–119 | 41–79 | 37–64 | 39–45 |
| Average longevity (days) | 116.2 | 65.5 | 55.3 | 41.3 |
| Range of total larval production (larvae/female) | 72–467 | 199–447.5 | 255–583 | 1–20 |
| Average larval production (larvae/female) | 195.13 | 316.75 | 480.25 | 3.88 |
| Reproduction frequency | 20 | 26 | 30 | 0.5 |
| Average clutch size | 9.58 | 12.18 | 16.00 | 7.75 |
| Hatching time (days) | 2.60 | 2.05 | 1.2 | 1.02 |

### TABLE 7
## Fecundity Life Table for *P. dubia* at Different Temperatures

| Age, x (days) | Survival Rate $l_x$ (°C) | | | | Birth Rate $m_x$ (°C) | | | | $l_xm_x$ (°C) | | | | $xl_xm_x$ (°C) | | | |
|---|---|---|---|---|---|---|---|---|---|---|---|---|---|---|---|---|
| | 20 | 25 | 30 | 35 | 20 | 25 | 30 | 35 | 20 | 25 | 30 | 35 | 20 | 25 | 30 | 35 |
| 1 | 1 | 1 | 1 | 1 | 0 | 0 | 0 | 0 | | | | | | | | |
| 3 | | 0.75 | 0.8 | 0.75 | 0 | 0 | 0 | 0 | | | | | | | | |
| 6 | 0.6 | | 0.68 | 0.45 | | 0 | 0 | 0 | | 0 | | | | 0 | | |
| 11 | 0.6 | 0.63 | 0.68 | 0.45 | 0 | 0 | 9.71 | 3 | 0 | 0 | 6.56 | 1.35 | 0 | 0 | 72.1 | 14.9 |
| 12 | 0.6 | 0.63 | 0.68 | 0.45 | 0 | 6.67 | 4.43 | 0 | 0 | 4.17 | 2.99 | 0 | 0 | 50 | 35.9 | 0 |
| 13 | 0.6 | 0.63 | 0.68 | 0.45 | 0 | 7.67 | 8.71 | 2 | 0 | 4.79 | 5.88 | 0.9 | 0 | 62.3 | 76.5 | 11.7 |
| 14 | 0.6 | 0.63 | 0.68 | 0.45 | 0 | 5.75 | 0 | 0 | 0 | 3.59 | 0 | 0 | 0 | 50.3 | 0 | 0 |
| 15 | 0.6 | 0.63 | 0.68 | 0.45 | 0 | 9.83 | 12.3 | 0 | 0 | 6.15 | 8.29 | 0 | 0 | 92.2 | 124 | 0 |
| 17 | 0.48 | 0.63 | 0.68 | 0.45 | 0 | 16.3 | 33.7 | 0 | 0 | 10.2 | 22.8 | 0 | 0 | 174 | 387 | 0 |
| 18 | 0.48 | 0.63 | 0.68 | 0.45 | 0 | 0 | 17.6 | 1 | 0 | 0 | 11.9 | 0.45 | 0 | 0 | 214 | 8.1 |
| 19 | 0.48 | 0.63 | 0.68 | 0.45 | 0 | 12.2 | 14.3 | 0 | 0 | 7.6 | 9.68 | 0 | 0 | 145 | 184 | 0 |
| 21 | 0.48 | 0.63 | 0.68 | 0.45 | 0 | 12.5 | 32.5 | 0 | 0 | 7.81 | 21.9 | 0 | 0 | 164 | 461 | 0 |
| 22 | 0.48 | 0.63 | 0.68 | 0.45 | 3.38 | 3.33 | 18.8 | 0 | 1.6 | 2.08 | 12.7 | 0 | 35.3 | 45.8 | 280 | 0 |
| 24 | 0.46 | 0.59 | 0.63 | 0.45 | 5.88 | 14.8 | 18.8 | 0 | 2.67 | 8.73 | 11.8 | 0 | 64.2 | 209 | 284 | 0 |
| 26 | 0.46 | 0.59 | 0.63 | 0.45 | 8.88 | 16.5 | 37.8 | 0 | 4.04 | 9.71 | 23.8 | 0 | 105 | 252 | 619 | 0 |
| 27 | 0.46 | 0.59 | 0.63 | 0.32 | 9.75 | 3.17 | 17.8 | 2 | 4.44 | 1.86 | 11.2 | 0.64 | 120 | 50.3 | 302 | 16.6 |
| 29 | 0.46 | 0.59 | 0.57 | 0.28 | 0 | 12.2 | 15.8 | 0 | 0 | 7.16 | 9.02 | 0 | 0 | 208 | 262 | 0 |
| 31 | 0.46 | 0.59 | 0.52 | 0.28 | 14.1 | 17.7 | 36.3 | | 6.43 | 10.4 | 18.7 | | 199 | 322 | 579 | |
| 33 | 0.46 | 0.59 | 0.52 | 0.24 | 11.6 | 10.3 | 18.5 | 0 | 5.29 | 6.08 | 9.54 | 0 | 175 | 201 | 315 | 0 |
| 35 | 0.46 | 0.55 | 0.52 | 0.2 | 6.38 | 15 | 19 | 0 | 2.9 | 8.27 | 9.8 | 0 | 102 | 290 | 343 | 0 |
| 37 | 0.46 | 0.55 | 0.46 | 0.12 | 9 | 17.3 | 25 | 0 | 4.1 | 9.56 | 11.5 | 0 | 152 | 354 | 424 | 0 |
| 39 | 0.46 | 0.48 | 0.46 | 0.1 | 6.38 | 13.5 | 19.7 | 0 | 2.9 | 6.45 | 9.01 | 0 | 113 | 252 | 351 | 0 |
| 41 | 0.46 | 0.48 | 0.46 | 0.05 | 13.5 | 15.6 | 12.7 | 0 | 6.15 | 7.46 | 5.8 | 0 | 252 | 306 | 238 | 0 |
| 43 | 0.46 | 0.44 | 0.39 | 0.05 | 8.5 | 13.6 | 20.7 | 0 | 3.87 | 6 | 8.12 | 0 | 166 | 258 | 349 | 0 |
| 45 | 0.46 | 0.44 | 0.39 | 0.03 | 5 | 4.4 | 22 | 0 | 2.28 | 1.94 | 8.64 | 0 | 102 | 87.4 | 389 | 0 |
| 47 | 0.46 | 0.37 | 0.33 | 0 | 10.6 | 15 | 17 | 0 | 4.84 | 5.52 | 5.56 | 0 | 227 | 259 | 262 | 0 |
| 49 | 0.46 | 0.29 | 0.33 | | 7.63 | 16 | 13.3 | | 3.47 | 4.71 | 4.36 | | 170 | 231 | 214 | |
| 50 | 0.46 | 0.29 | 0.33 | | 2.63 | 8.4 | 5.33 | | 1.2 | 2.47 | 1.75 | | 59.8 | 124 | 87.3 | |
| 52 | 0.46 | 0.29 | 0.33 | | 0 | 13 | 9.67 | | | 3.82 | 3.16 | | | 199 | 165 | |
| 53 | 0.46 | 0.24 | 0.26 | | 10.3 | 7.8 | 6 | | 4.67 | 1.86 | 1.57 | | 247 | 98.8 | 83.3 | |
| 56 | 0.46 | 0.24 | 0.26 | | 6.25 | 15 | 14.3 | | 2.85 | 3.59 | 3.75 | | 159 | 201 | 210 | |
| 59 | 0.46 | 0.24 | 0.2 | | 13 | 25.5 | 18 | | 5.92 | 6.09 | 3.53 | | 349 | 360 | 209 | |
| 61 | 0.46 | 0.24 | 0.13 | | 4 | 5.33 | 3 | | 1.82 | 1.28 | 0.39 | | 111 | 77.8 | 24 | |
| 64 | 0.46 | 0.24 | 0 | | 7.67 | 7.33 | 0 | | 3.49 | 1.75 | 0 | | 223 | 112 | 0 | |
| 66 | 0.44 | 0.21 | | | 6.5 | 7.33 | | | 2.83 | 1.53 | | | 187 | 101 | | |
| 68 | 0.42 | 0.12 | | | 3.88 | 3.67 | | | 1.61 | 0.44 | | | 110 | 29.8 | | |
| 70 | 0.42 | 0.09 | | | 5.13 | 3 | | | 2.15 | 0.27 | | | 151 | 18.8 | | |
| 71 | 0.4 | 0.03 | | | 4 | 0.33 | | | 1.6 | 0.01 | | | 114 | 0.71 | | |
| 74 | 0.4 | 0.03 | | | 8.75 | 2.5 | | | 3.5 | 0.08 | | | 259 | 5.53 | | |
| 77 | 0.4 | 0.03 | | | 7 | 0 | | | 2.8 | 0 | | | 216 | 0 | | |
| 79 | 0.4 | 0 | | | 2.63 | 0 | | | 1.05 | 0 | | | 83 | 0 | | |
| 84 | 0.36 | | | | 19.9 | | | | 7.15 | | | | 601 | | | |
| 89 | 0.34 | | | | 10.9 | | | | 3.69 | | | | 329 | | | |
| 91 | 0.34 | | | | 10.5 | | | | 3.57 | | | | 325 | | | |
| 94 | 0.32 | | | | 6.83 | | | | 2.19 | | | | 206 | | | |
| 98 | 0.32 | | | | 7.33 | | | | 2.35 | | | | 230 | | | |
| 103 | 0.32 | | | | 10.7 | | | | 3.41 | | | | 352 | | | |
| 104 | 0.32 | | | | 2.5 | | | | 0.8 | | | | 83.2 | | | |
| 105 | 0.28 | | | | 0 | | | | 0 | | | | 0 | | | |
| 106 | 0.28 | | | | 1 | | | | 0.28 | | | | 29.7 | | | |

*Table 7 continues*

**TABLE 7 (continued)**

| | | | | | | | |
|---|---|---|---|---|---|---|---|
| 108 | 0.28 | 2.8 | 0.78 | | | | 84.7 |
| 110 | 0.28 | 1.6 | 0.45 | | | | 49.3 |
| 112 | 0.28 | 7.2 | 2.02 | | | | 226 |
| 114 | 0.28 | 6 | 1.68 | | | | 192 |
| 117 | 0.14 | 0 | 0 | | | | 0 |
| 118 | 0.08 | 0 | 0 | | | | 0 |
| 119 | 0.05 | 0 | 0 | | | | 0 |
| 120 | 0 | 0 | 0 | | | | 0 |
| Total $R_0$ | | | 119 | 163 | 264 | 3.3 | |

**TABLE 8**
**Life Table Parameters of *P. dubia* at Different Temperatures**

| Growth Parameters | Temperature (ºC) | | | |
|---|---|---|---|---|
| | 20 | 25 | 30 | 35 |
| Net reproduction rate ($R_0$) | 119 | 163 | 264 | 3.3 |
| Population growth cycle (T) | 58.5 | 30.4 | 28.6 | 15.36 |
| Intrinsic growth rate ($r_m$) | 0.082 | 0.168 | 0.195 | 0.079 |
| Population doubling time (t) | 8.49 | 4.13 | 3.56 | 8.833 |
| Finite growth rate ($\lambda$) | 1.085 | 1.183 | 1.215 | 1.082 |

### Effects of Temperature on Generation Survival Curve

The generation survival curve of *P. dubia* was steepest and shortest at 35°C (Fig. 6), but was similar between 25 and 30°C, and longest at 20°C, which corresponded to their life cycle.

## DISCUSSION

### Effects of Temperature on Juvenile Development and Survival

Water temperature is an important factor that affects the survival, development, and growth of aquatic invertebrates[5,13]. Temperature affects metabolism level and oxygen consumption that, in turn, affect an animal's ability to resist environmental changes. High temperatures can accelerate metabolism and oxygen consumption, leading to excessive use of protein and a low survival rate[14,15,16]. This is in agreement with our findings that indicate significant effects of temperature on development time, survival rate, and growth rates of *P. dubia*. As the temperature increases, the development time required for each larval stage decreases and growth rates increase. The survival rate increases as the temperature increases within the range from 15 to 30ºC. However, when the temperature exceeds 35ºC, the larval survival rate begins to decline and larval development slows down as the temperature drops. During copepod development, there are three critical stages, i.e., fertilized egg to nauplii, NIII to NIV, and NVI to CI, which are extremely sensitive to environmental changes as evidenced by high mortality. These periods will be prolonged if development slows down, leading to high mortality. At a temperature of 15ºC, the entire larval development time is 33 days. This explains the high mortality under low temperatures, which delay larval development significantly. Therefore, temperature is a critical factor that affects the copepod life cycle.

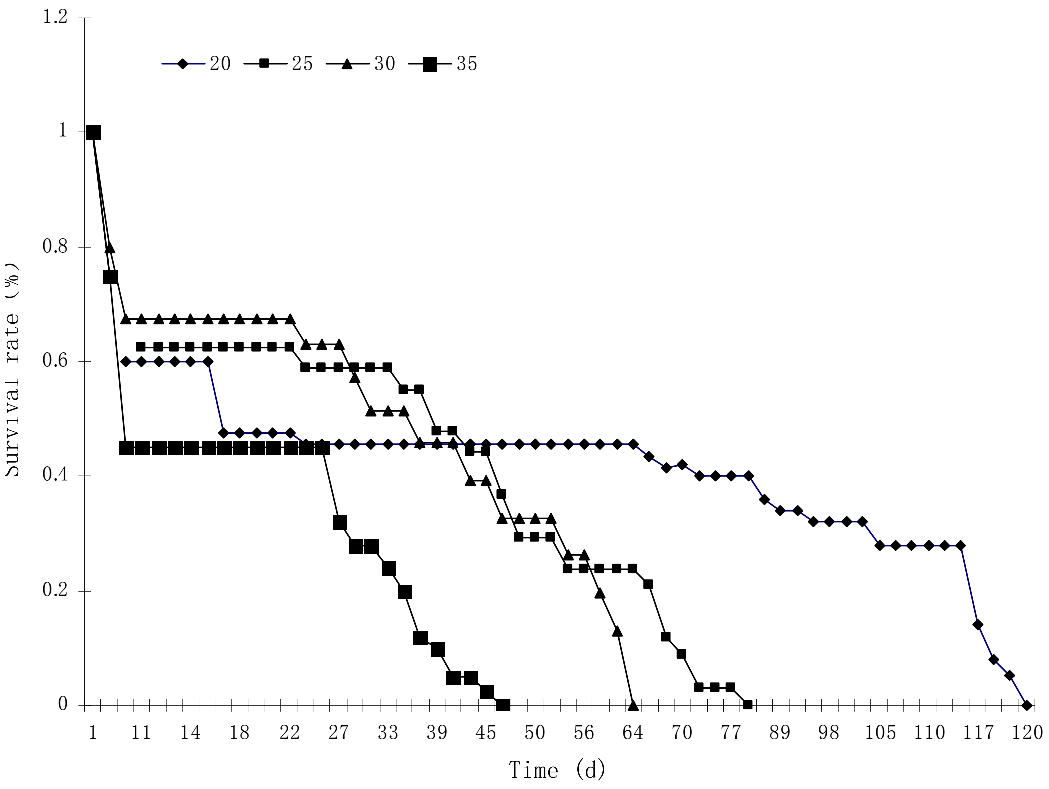

**FIGURE 6**. Survival curves of *P. dubia* at different temperatures.

Within the experimental range of temperature, there was no difference in body weight between nauplii and copepodites. Previous studies found that adult body weight can attain its maximum under low temperatures[17] and large individuals require a longer period of development[16]. This finding is contradictory to our results, which revealed no significant difference in body length for the same larval stage under different temperatures. We also did not observe an increase in body length with a decrease in water temperature in our experiment.

### Effects of Temperature on Reproduction

Chen[18] reported an increase in mating frequency under high temperatures. However, negative effects on the reproduction of copepods have also been reported. For example, Li and Chen[19] indicated in their experiment that the egg production increased as the temperature increased and reached its peak at 24ºC, and then decreased with further increase in temperature. McKinnon[20] also found that the body length of a subtropical copepod, *Acrocalanus gibber*, increased with water temperature. These experiments indicate that an optimal spectrum of temperature is species specific[14]. This is in agreement with our results that indicate the importance of temperature to the longevity and reproduction of *P. dubia*. As temperature increases, the longevity and hatching time for *P. dubia* decrease. Total egg production and mating rate increase as temperature increases, but decreases when the temperature exceeds 30ºC. Hatching rate and reproduction frequency are at the lowest at 35ºC. Therefore, within a suitable temperature range, elevated temperature may promote copepod reproduction, increase egg production, and shorten reproduction cycle and hatching time.

Chen[18] found that at high temperatures, mating individuals increased and mating time decreased. Our experiment indicates that the optimal temperature ranges from 25 to 30ºC for population growth and reproduction by *P. dubia.* Intrinsic, finite, and net growth rates are the highest at 30ºC. Meanwhile, the individual development and reproduction (clutch size and hatching rate) are also high. At 30°C, it takes 210 h or 8.75 days for NII to reach the adult stage, with a survival rate of 80%, an average longevity of 55.25 days, an average total hatching number of 480.21/female, and a reproduction frequency of 30 with an average hatching of 16 larvae. Under the optimal temperature of 30ºC, the net production rate ($R_0$) is 264, generation time (T) is 28.6 days, intrinsic growth rate ($r_m$) is 0.195, finite growth rate ($\lambda$) is 1.215, and population doubling time (t) is 3.55. These data suggest a very high reproduction capacity of *P. dubia* under the optimal temperature and food conditions.

Banse[21] found that intrinsic growth rate varied among different sizes of invertebrates at the same temperature. Ji et al.[22] found intrinsic growth rate of acarus (*Oligonychus biharensis* Hirstwas) at 35ºC also higher than at 25ºC, but net reproduction rate and average egg production at 35ºC were lower than at 25ºC, which led to a conclusion that 25ºC is a more suitable temperature for *O. biharensis*. Our results show little difference in intrinsic growth rate at 35ºC (0.79) and 20ºC (0.082). However, this does not imply that there was no difference in population growth between these temperatures. At 35ºC, the reproduction rate of the next generation is 3.875 (Table 8), while the net reproduction rate at 20ºC is 119; the average hatching rate is 3.875 larvae/female at 35ºC, which is significantly lower than the 195.125 larvae/female at 20ºC. It is concluded that population reproduction and growth are significantly better at 20ºC than at 35ºC.

### ACKNOWLEDGMENTS

This study was funded by the Ministry of Science and Technology of China (2006GB2E000224) and Guangdong Provincial Science and Technology Projects (2006A20305003).
